# Muscarinic cholinergic receptor (M_2_) plays a crucial role in the development of myopia in mice

**DOI:** 10.1242/dmm.010967

**Published:** 2013-05-02

**Authors:** Veluchamy A. Barathi, Jia Lin Kwan, Queenie S. W. Tan, Sung Rhan Weon, Li Fong Seet, Liang Kee Goh, Eranga N. Vithana, Roger W. Beuerman

**Affiliations:** 1 Singapore Eye Research Institute, 11 Third Hospital Avenue, 168751 Singapore, Singapore; 2 Department of Ophthalmology, Yong Loo Lin School of Medicine, National University of Singapore, 119074 Singapore, Singapore; 3 Duke-National University of Singapore Graduate Medical School, 8 College Road, 169857 Singapore, Singapore; 4 Department of Epidemiology and Public Health, Yong Loo Lin School of Medicine, National University of Singapore, 119074 Singapore, Singapore

## Abstract

Myopia is a huge public health problem worldwide, reaching the highest incidence in Asia. Identification of susceptible genes is crucial for understanding the biological basis of myopia. In this paper, we have identified and characterized a functional myopia-associated gene using a specific mouse-knockout model. Mice lacking the muscarinic cholinergic receptor gene (*M_2_*; also known as *Chrm2*) were less susceptible to lens-induced myopia compared with wild-type mice, which showed significantly increased axial length and vitreous chamber depth when undergoing experimental induction of myopia. The key findings of this present study are that the sclera of *M*_2_ mutant mice has higher expression of collagen type I and lower expression of collagen type V than do wild-type mice and mice that are mutant for other muscarinic subtypes, and, therefore, *M*_2_ mutant mice were resistant to the development of experimental myopia. Pharmacological blockade of M_2_ muscarinic receptor proteins retarded myopia progression in the mouse. These results suggest for the first time a role of *M*_2_ in growth-related changes in extracellular matrix genes during myopia development in a mammalian model. M_2_ receptor antagonists might thus provide a targeted therapeutic approach to the management of this refractive error.

## INTRODUCTION

Myopia is the most common human ocular disorder. In the United States, one third of the adult population has some degree of myopia (Vitale et al., 2008). The prevalence of myopia varies across different populations, with the highest prevalence observed among Asians in countries such as Singapore, China, Taiwan and Japan (Lin et al., 1988; Chow et al., 1990; Saw et al., 1996; Wong et al., 2000; Seet et al., 2001; Wong and Saw, 2004; Saw et al., 2008; Dirani et al., 2010; Pan et al., 2011). In addition to the associated substantial visual loss and economic burden, high myopia [spherical equivalent >6 diopter (D)] has been associated with degenerative diseases such as myopic macular degeneration, retinal detachment and posterior staphyloma (Hotchkiss and Fine, 1981; Metlapally et al., 2008). These lifelong disease issues make myopia a long-term economic and social burden on the health care system (Curtin, 1985; Javitt and Chiang, 1994; Vitale et al., 2006).

Axial elongation of the posterior chamber of the eye is the phenotypic hallmark of myopia and causes images to focus in front of the retina (McBrien and Gentle 2003; Morgan et al., 2012). This could also be due to uncompensated growth of the fibrous outer coat of the eye, the sclera (McBrien and Gentle, 2001). Histopathological and biochemical studies have shown that weakening of the scleral matrix, probably through modulation of proteoglycan synthesis by scleral fibroblasts (SFs), can lead to scleral thinning and axial elongation in myopia (Rada et al., 2006; Qu et al., 2006; Siegwart and Strang, 2007; McBrien et al., 2009a). Although a detailed biological mechanism underlying myopia has not been described yet, several studies have suggested changes in collagen levels as an important contributor to the disease pathology and progression (and Gentle et al., 2003; Zhao et al., 2012; Zhou et al., 2012). Therefore, investigation of scleral remodeling and the mechanism involved in SF cell proliferation have been considered appropriate systems to discover useful anti-myopic drugs.

In myopic children, muscarinic antagonists such as atropine and pirenzepine have been used therapeutically to slow the progression of disease (Saw et al., 2002; Tan et al., 2005; Chua et al., 2006; Siatkowski et al., 2008; Gwiazda, 2009; Tong et al., 2009; Ganesan and Wildsoet, 2010). Atropine, a non-subtype-selective muscarinic-receptor blocker has been shown to be effective at a concentration of 0.025% (Fang et al., 2010), as compared with pirenzepine, which shows a limited degree of selectivity for blocking only M_1_ receptors at 2% concentration (Siatkowski et al., 2008). There are five muscarinic receptor subtypes (M_1_–M_5_), which are all ubiquitously expressed in the eye, including in SFs, of humans and mice (Friedman et al., 1988; Qu et al., 2006; Liu et al., 2007a; Barathi et al., 2009a) (supplementary material Table S1). It is well known that atropine affects all subtypes of the muscarinic receptor gene family, M_1_–M_5_ (Hulme et al., 1990; Caulfield and Birdsall, 1998; Luft et al., 2003). These receptor subtypes are well represented in the eye and neither atropine nor pirenzepine are specific inhibitors for myopia. Therefore, it is not clear as to which muscarinic receptor subtype plays a major functional role in myopia pathology.

Previous studies from our laboratory and others have demonstrated the ability to induce myopia in mice (Schaeffel et al., 2004; Faulkner et al., 2007; Barathi et al., 2008), and now, the availability of selective M_1_–M_5_ muscarinic cholinergic receptor knockout animals (Wess et al., 2007) will enable us to decipher the role of specific muscarinic receptor subtypes in the development of myopia. In this study, we report a crucial role of the M_2_ receptor (encoded by *Chrm2*, which is referred to here as *M_2_*) in the development of myopia.

**TRANSLATIONAL IMPACT**

**Clinical issue**

Myopia, or near-sightedness, is the most common human eye disorder. The condition results from axial elongation of the eye, primarily in the posterior segment, which causes images to focus in front of the retina instead of directly on it. Axial elongation is thought to be induced by remodeling of the sclera, the extracellular-matrix-rich outer shell of the eye. Image defocus in the myopic eye can be easily corrected by a variety of refractive methods, including lens usage or corneal surgery. However, patients remain at increased risk of developing cataracts, open angle glaucoma and, more seriously, retinal detachment or blindness. Therefore, it is of crucial importance to understand the underlying mechanisms and develop new therapeutic approaches that stop or reduce the progression of myopia at the molecular level. Evidence from animal studies indicates a role for muscarinic cholinergic receptors in myopia pathology and, in line with this, clinical trials have demonstrated the efficacy of muscarinic antagonists in retarding childhood myopia. However, the mechanistic details, including the relative importance of different muscarinic receptor subtypes (M_1_–M_5_) in myopia development, remain poorly understood.

**Results**

The authors previously established a mouse model of experimental myopia characterized by axial elongation and sclera remodeling, thus mimicking human myopia. Exploiting this model, the group sought to determine the effects of inactivating each of the muscarinic receptor subtypes on the susceptibility of mice to develop myopia. They found that mutant mice lacking the M_2_ receptor encoded by the *Chrm2* gene (referred to here as *M_2_*) are less susceptible to the induction of experimental myopia than are wild-type mice and mice lacking the M_1_, M_4_ or M_5_ subtypes. M_3_ receptor mutants were also found to be relatively resistant to the induction of myopia. Previous studies have implicated changes in collagen levels as an important contributory factor to scleral remodeling and axial elongation, prompting the authors to investigate collagen expression in the mutant mice. Choosing *M_2_* knockout mice for further analysis, they show that these mice express higher levels of type I collagen and lower levels of type 5 collagen compared with wild-type mice. Furthermore, the authors examined the effects of *M_2_* siRNA knockdown on scleral fibroblast cell proliferation *in vitro*; this analysis revealed that scleral fibroblast growth is hindered by loss of M_2_. Finally, they demonstrate that specific pharmacological blockade of M_2_ muscarinic receptors inhibits the progression of myopia.

**Implications and future directions**

This study provides *in vivo* evidence to support an important role for the M_2_ muscarinic receptor in myopia development. The data indicate that the actions of the M_2_ receptor are mediated by changes in the expression of key extracellular matrix proteins, linking the functional role of M_2_ with scleral remodeling in myopia. The authors’ pharmacological analysis suggests that specific M_2_ receptor antagonists could provide a targeted therapeutic approach for the treatment of myopia and its associated conditions. The study also highlights the utility of the mouse as a model for myopia, particularly in conjunction with new technologies that can measure ocular dimensions and optical properties with high precision. Further mouse studies are needed to pinpoint and validate the downstream targets of M_2_ and to investigate the role of the M_3_ receptor subtype in myopia development.

## RESULTS

### Development of myopia in muscarinic receptor mutant mice

**Fig. 1.**
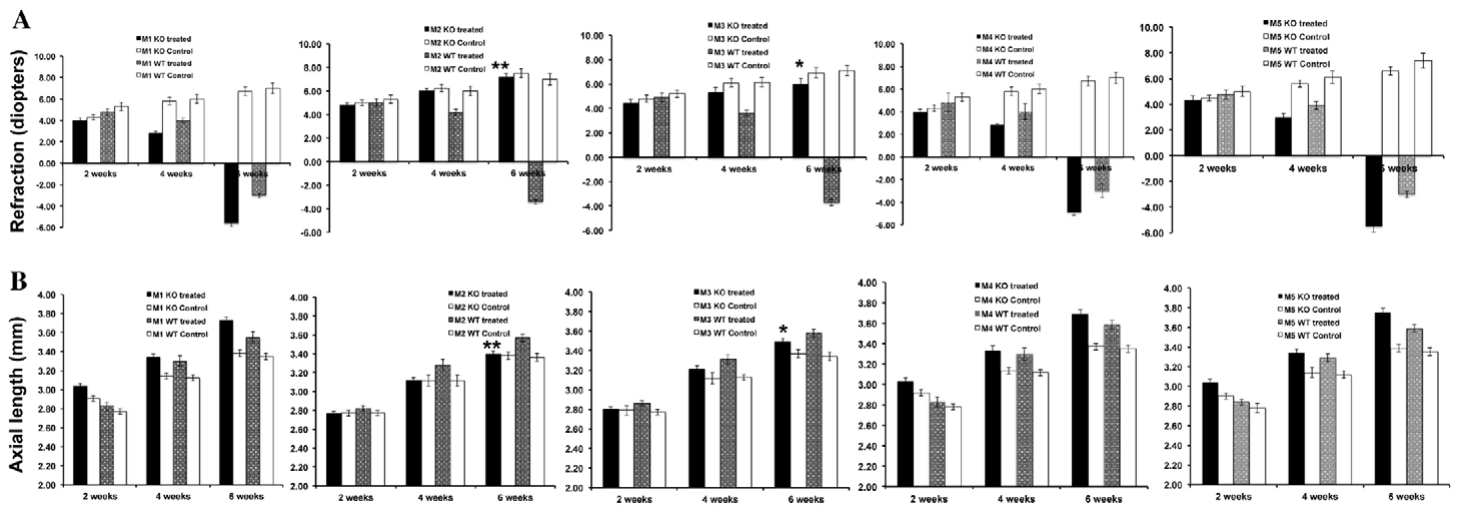
**The induction of myopia in muscarinic-receptor-knockout mice (*M_1_–M_5_*).** Myopia induction was performed using a uniocular −15 diopter negative lens in wild-type (WT) and homozygous *M_1_*–*M_5_* mutant mice. Results at 2, 4 and 6 weeks are shown. The axial length measurements (B) were measured using the OLCI-AcMaster (*in vivo*: accuracy ± 10 μm) and refraction (diopters; A) was measured by automated infrared photorefractor at 2, 4 and 6 weeks after induction of myopia. (A) Measurements of refractive state of muscarinic receptor mutant and WT mice. (B) *In vivo* measurement of axial length of myopic muscarinic receptor mutant and WT mice. Data are represented as mean ± s.d.; **P<0.001; *P<0.05.

A spectacle lens (−15 D) was placed over the right eye of the muscarinic receptor mutant mice and wild-type (WT) mice to induce myopia. Left eyes were uncovered to serve as experimental controls (*n*=50 mice in each strain). The refractive state and axial length of the myopic and control eyes were measured at 2, 4 and 6 weeks after lens treatment. Measurements were used to compare the degree of myopia between the control and lens-treated eyes. In addition, the degree of myopia was also compared between mice that were mutant for the different muscarinic receptors (M_1_–M_5_). Following lens treatment, lens-treated eyes had developed myopia after 6 weeks, whereas, with the exception of *M_2_* and *M_3_* (*Chrm3*) mutant mice, untreated control eyes had not (Fig. 1). Comparison between muscarinic receptor mutants showed that *M_1_* (*Chrm1*), *M_4_* (*Chrm4*) and *M_5_* (*Chrm5*) mutants had significantly higher myopia in the lens-treated eyes than did *M_2_* and *M_3_* mutants. This result was similar for both the refractive state (Fig. 1A) and axial length (Fig. 1B) measurements. However, *M_2_* mutants showed no significant increase between lens-treated eyes and control eyes.

### Role of M_2_ in myopia

We found that *M_2_* mutant mice were resistant to the standard methods for inducing experimental myopia and these treatments were not successful in developing a myopic refraction or increasing axial length. After application of a −10 and −15 D negative lens as one of the standard methods for induction of myopia in mice (Barathi et al., 2008), *M_2_* mutant mice remained hyperopic at week 8 (6 weeks after induction) compared with WT mice (*P*<0.07 at 2 weeks induction, *P*<0.05 at 4 weeks induction and *P*<0.01 at 6 weeks induction, *n*=50; supplementary material Fig. S1A). Similarly, ocular light diffuser treatment (which prohibits sharply focused retinal images) in *M_2_* mutant mice was not effective in producing either structural or refractive changes, whereas the WT mice responded as before (data not shown). Importantly, a plano lens of the same material did not induce myopia in WT mice (Barathi et al., 2008). Axial length increased significantly in negative-lens-treated WT mice at week 8 (6 weeks after induction; *P*<0.001), but not in *M_2_* mutant mice (*P*=0.11 at 2 weeks induction; *P*=0.14 at 4 weeks induction; *P*=0.12 at 6 weeks induction; *n*=50; supplementary material Fig. S1B), compared with contralateral uncovered eyes of the same animals. These results indicated that M_2_ plays a crucial role in the development of myopia in the mouse model. There were no significant differences in corneal thickness or anterior chamber depth between minus-lens-treated eyes and contralateral eyes in both *M_2_* mutant and WT mice (supplementary material Fig. S1C,D). The increase in lens thickness (supplementary material Fig. S1E) and vitreous chamber depth (supplementary material Fig. S1F) were statistically significant in minus-lens-treated WT eyes after 4 weeks of induction (*P*<0.06 at 2 weeks induction; *P*<0.05 at 4 weeks induction; *P*<0.01 at 6 weeks induction; *n*=50) compared with contralateral eyes. However, this was not significant in myopic-induced *M_2_* mutant mice when comparing with contralateral eyes (*P*=0.15, *n*=50).

### Expression of M_2_ in mouse scleral tissue

Recent studies have demonstrated that all five types of muscarinic receptors are found in tree shrew, mouse and human SFs (McBrien et al., 2009b; Barathi et al., 2009a). In our recent report, we observed that cultured mouse and human SFs express M_1_–M_5_ protein and *M_1_*–*M_5_* mRNA (Barathi et al., 2009a). The protein for the M_2_ receptors was found to be expressed in sclera from naive (non-myopic) WT (*M_2_*^+/+^) mice and heterozygous *M_2_*^+/−^ mutant mice, but not in homozygous (*M_2_*^−/−^) mutant mouse sclera (supplementary material Fig. 2A–C).

**Fig. 2.**
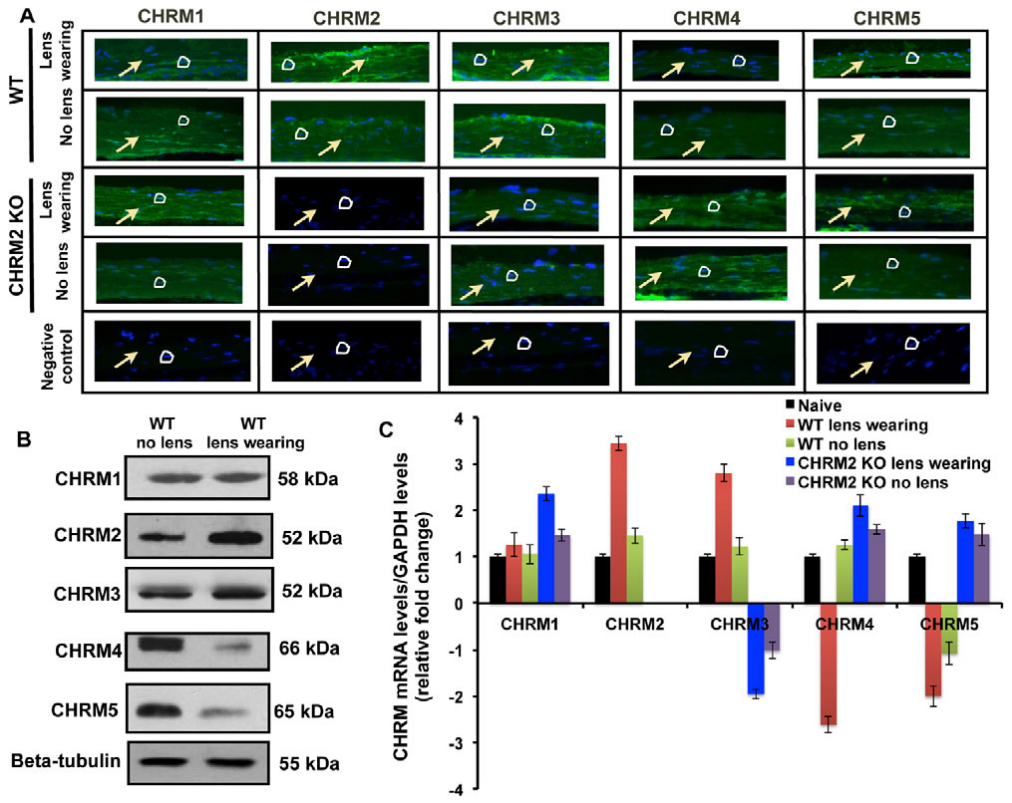
**Muscarinic receptor expression in 8-week-old (6 weeks after induction of myopia) myopic and control scleral tissue.** (A) Immunofluorescent staining images using primary antibody against M_1_–M_5_ in 8-week-old (6 weeks after induction of myopia) minus-lens-induced WT and *M_2_* (*Chrm2*)-mutant mice scleral tissue. Arrows indicate scleral fibroblast and circles represent the nucleus. The minus-lens-induced WT sclera shows higher expression of M_2_ as compared with the control sclera. However, M_4_ and M_5_ levels were downregulated, and there was no significant difference in M_1_ expression. High expression of M_1_, M_4_ and M_5_ and low expression of M_3_ was detected in the minus-lens-induced *M_2_*-mutant mice when compared with naive sclera, and no expression was detected for *M_2_*. The sections were incubated with 2% goat serum and labeled with FITC secondary antibody (control). DAPI was used to stain nuclei. Magnification at 400×. (B) Western blot image showing M_1_–M_5_ (CHRM1-CHRM5) protein and tubulin loading control in WT murine sclera treated with minus lens and control. The M_2_ protein was upregulated in the WT myopic sclera as compared with the control sclera. (C) qRT-PCR showing relative *M_1_*–*M_5_* transcript levels in *M_2_*^−/−^ and WT mice sclera. Transcript levels were normalized to *GAPDH* mRNA expression. The −10 D lens-treated WT scleral *M_2_* and *M_3_* mRNA levels were upregulated as compared with naive sclera. However, *M_4_* and *M_5_* mRNA levels were downregulated, and there was no significant difference in *M_1_* mRNA level. The mRNA level of *M_1_*, *M_4_* and *M_5_* was upregulated, and *M_3_* was downregulated in the minus-lens-induced *M_2_*-mutant mice when compared with naive sclera, and no expression was detected for *M_2_*. Data are represented as mean ± s.d.; *P<0.05 and **P<0.01.

### Upregulation of *M_2_* in mouse myopic sclera

Immunohistochemistry and western blotting studies showed that M_2_ receptor protein expression was significantly increased in the WT myopic sclera as compared with control sclera and sclera from mutants of other muscarinic receptor subtypes (Fig. 2A,B). Similarly, quantitative real-time polymerase chain reaction (qRT-PCR) showed that *M_2_* transcript levels were upregulated in WT myopic sclera compared with control sclera and sclera from mutants of other muscarinic receptor subtypes (Fig. 2C). As expected, no *M_2_* mRNA was detected in sclera from *M_2_* mutant mice. *M_1_*, *M_4_* and *M_5_* transcript levels were upregulated (*P*=0.023, 0.001 and 0.0002, respectively), and *M_3_* mRNA level was downregulated (*P*=0.0004) in *M_2_* mutant mice sclera.

### Collagen expression in *M_1_*–*M_5_* mutant mouse sclera

**Fig. 3.**
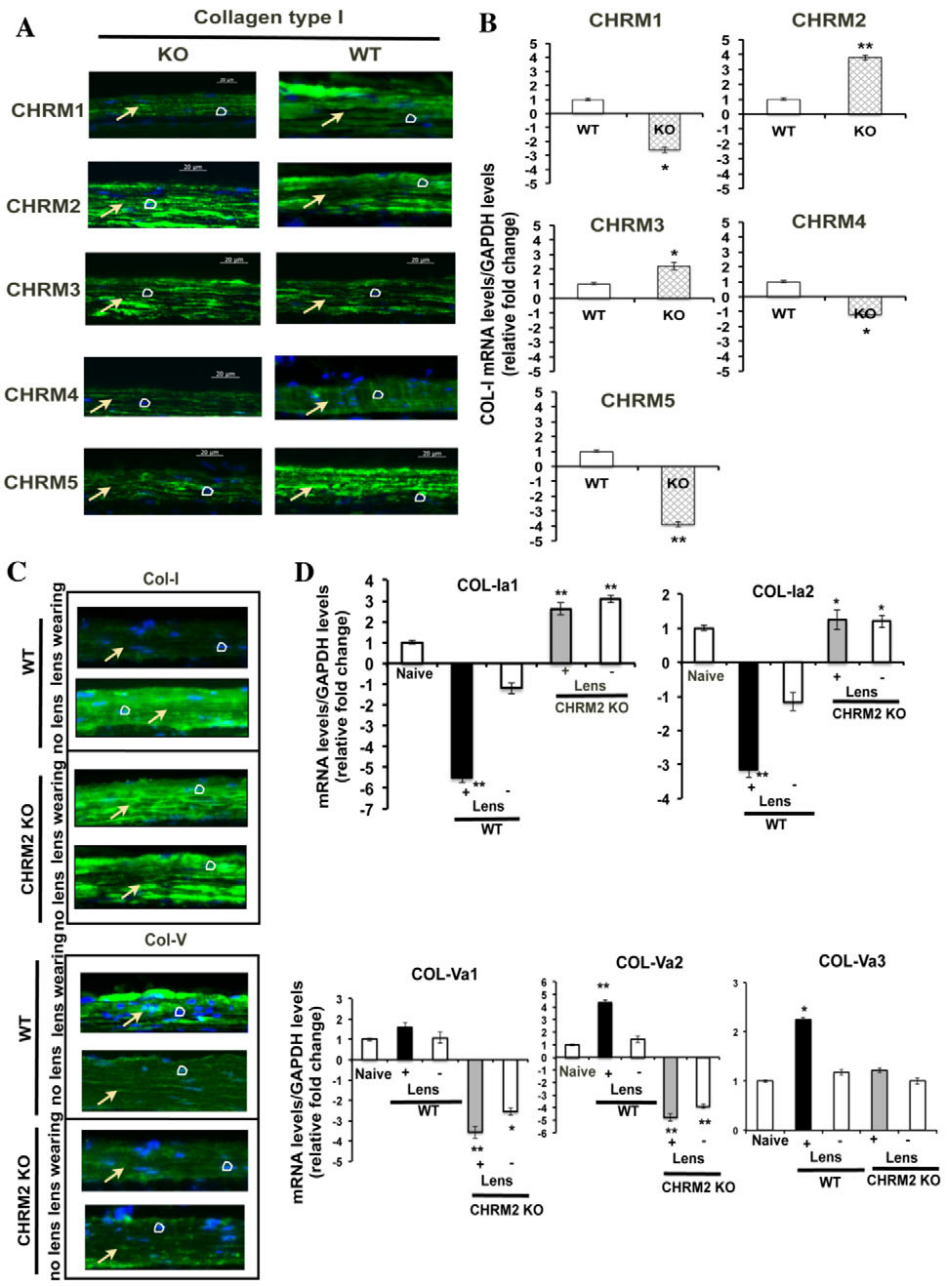
**Expression of collagens in *M*_1_*M_5_* (*Chrm1-Chrm5*)-mutant mice and myopic sclera.** (A) Immunofluorescent staining images using primary antibody against COL-I in 8-week-old WT and *M_1_–M_5_*-mutant mice scleral tissue. Arrows indicate sclera and circles represent nucleus. Scale bars: 20 μm. The *M_2_*-mutant mice sclera shows higher expression of COL-I as compared with the WT and other subtype sclera. (B) The mRNA level of collagen 1a1 in *M_1_*–*M_5_* mutant mice sclera was compared with WT naive sclera after normalization with *GAPDH* reference gene. *COL-I* was significantly upregulated in *M_2_*- and *M_3_*-mutant mice sclera and this was significantly downregulated in the *M_1_*-, *M_4_*- and *M_5_*-mutant mice when compared with WT sclera. Data are represented as mean ± s.d.; *P<0.05 and **P<0.01. (C) Type I collagen expression was reduced in WT minus-lens-induced sclera compared with control and *M_2_*-mutant minus-lens-induced sclera. This was reversed in collagen type V expression. Arrows indicate sclera and circles represent nucleus. (D) The mRNA level of collagen 1a1, 1a2, 5a1, 5a2 and 5a3 in WT and *M_2_*-mutant lens-induced (+ Lens; −15 D lens wearing eyes) and control (– Lens; uncovered eyes) mouse sclera was compared with naive sclera after normalized with *GAPDH* reference gene. Collagen 1a1 and 1a2 was downregulated in the WT myopic sclera and collagen 5a1, 5a2 and 5a3 was upregulated. This was reversed in minus-lens-wearing *M_2_*-mutant sclera. Data are represented as mean ± s.d.; *P<0.05 and **P<0.01.

Our study results confirm that *M_2_* mutant mice are resistant to induced experimental myopia. Earlier studies reported that collagen type I (COL-1) was significantly reduced in tree shrew and human myopic sclera (Norton and Rada, 1995; Avetisov et al., 1983). Hence, we investigated the underlying changes in collagen in myopia development. The present study results show that *M_2_* mutant mice sclera has higher expression of COL-1 than WT and other subtype mutant mice in cellular and mRNA levels (Fig. 3A,B; *n*=3 independent samples; *P*=0.00003), and, therefore, *M_2_* mutant mice were resistant to the development of experimental myopia. This result also suggests that muscarinic receptors might have a role in collagen production in mammalian sclera.

We also determined the expression of *COL-1* and collagen type V (*COL-5*) in WT and *M_2_* mutant minus-lens-induced myopic sclera. *COL-1* expression was significantly reduced in WT myopic sclera compared with *M_2_* mutant and control sclera groups (Fig. 3C). However, the expression pattern for *COL-5* was reversed. The mRNA expression of collagen genes was investigated in WT and *M_2_* mutant lens-induced sclera to confirm the scleral remodeling process in myopic sclera. The transcript level of *COL-1A1* and *COL-1A2* was significantly downregulated in the WT myopic sclera as compared with naive, control and *M_2_* mutant groups (Fig. 3D; *n*=3; *P*<0.00001). However, the mRNA expression of *COL-5A2* and *COL-5A3* was significantly upregulated in the myopic sclera and no significant difference was found in the *COL-5A1* mRNA level.

### SF cell growth controlled by M_2_

**Fig. 4.**
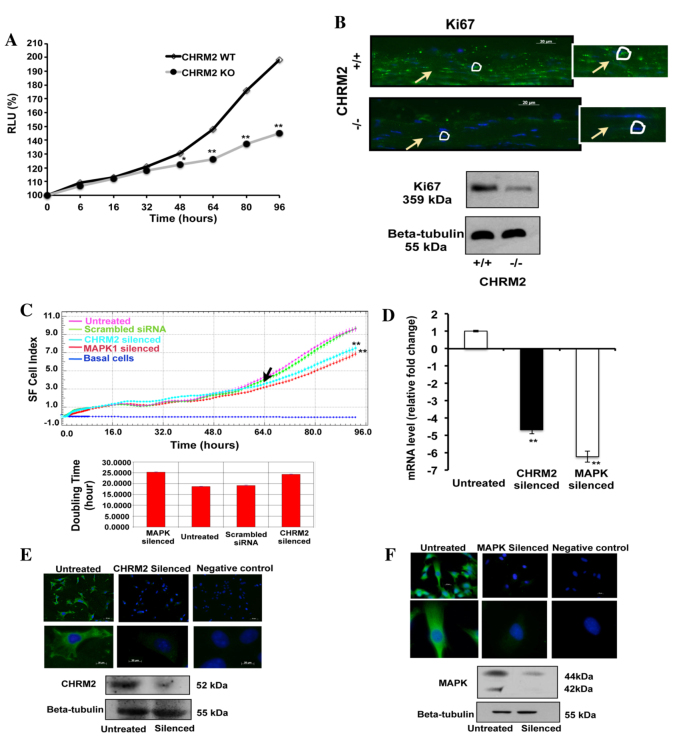
**Role of *M_2_* (*Chrm2*) in SF cell proliferation.** (A) SF cell proliferation in WT and *M_2_*-mutant mice is illustrated on the graph. BrdU incorporation after 6, 16, 32, 48, 64, 80 and 96 hours in SFs from WT and *M_2_*-mutant mice were measured by ELISA to investigate the role of *M_2_* in SF cell proliferation. Proliferation of *M_2_*-mutant SFs was significantly inhibited in a time-dependent manner (*P*<0.05, ANOVA, *n*=3) and this inhibition was increased in *M_2_* WT mice (*P*<0.05, ANOVA, *n*=3). Data are represented as mean ± s.e.m. *P<0.05 and **P<0.01 versus WT SF. (B) Immunofluorescent staining images using primary antibody against Ki67 (cell proliferation marker) in WT and *M_2_*-mutant mice scleral tissue. Arrows indicate SF and circles represent nucleus. The *M_2_*-mutant mice SF shows significantly lower expression of Ki67 as compared with the WT SF. Scale bars: 20 μm; inset shows enlarged images, magnification 400×. Experiment was performed in triplicates. (C) Effect of *M_2_* siRNA knockdown on SF cell growth. Cell proliferation was significantly reduced in the *M_2_*-silenced mouse SF cells as compared with WT SF cells. Cell growth of *M_2_*-silenced cells as compared with WT cells was observed over a growth period of 96 hours after seeding at a concentration of 3500 cells per 96 well. Passage 4 cells were used. The *M_2_*-silenced cell growth was reduced at 64 hours (arrow) compared with controls, and this difference was more significant as time increased. Pink line: WT untreated; green line: WT cells treated with scrambled siRNA (negative control); cyan line: siRNA *M_2_* (target gene); red line: siRNA *MAPK1* (positive control); blue line: basal cells (blank). Data are represented as mean ± s.d. **P<0.01. Doubling time for the various types of transfected mouse SFs in the log phase of growth is illustrated in the bottom graph. It was shown that both the untreated and negative controls have a lower/shorter doubling time as opposed to *MAPK1*- and *M_2_*-silenced cells (a shorter doubling time equates to faster growth). It is expected that a knockdown of the *MAPK1* gene, whose functions include an involvement in development and cell proliferation, will result in a slowdown in cell proliferation. An increase in doubling time was observed for *M_2_*-knockdown mouse SFs, which could be an indication that M_2_ knockdown, either directly or indirectly via interacting with other genes, results in a decrease in cell proliferation ability. (D) The gene expression profile of *M_2_* and *MAPK* after they were silenced by their respective siRNAs for 3 days is shown. The gene expressions were normalized with *GAPDH* reference gene. MAPK was used as a positive control. *M_2_* and *MAPK* mRNA level was significantly downregulated after 3 days of treatment with their respective siRNAs, compared with controls. (E) Images of mouse SFs, treated and untreated, taken using a fluorescence microscope (Axiophan 2). Untreated SFs stained with anti-M_2_ primary antibody; silenced treated with *M_2_* siRNA (Qiagen); negative control for M_2_ stained only with goat anti-rabbit secondary antibody (Pierce Biotechnology). Upper panel magnification, 20×. Lower panel magnification, 40×. Experiment was performed in triplicates. Western blot confirms the same pattern of expression and β-tubulin was used as a loading control; untreated: control; silenced: siRNA *M_2_*. (F) Images of mouse SFs, treated and untreated, taken using a fluorescence microscope (Axioplan 2). Untreated SFs stained with anti-MAPK primary antibody; silenced treated with *MAPK* siRNA (Qiagen); negative control for MAPK stained only with goat anti-rabbit secondary antibody (Pierce Biotechnology). Upper panel magnification, 20×. Lower panel magnification, 40×. Experiment was performed in triplicates. Western blot confirms the same pattern of expression and β-tubulin was used as a loading control; untreated: control; silenced: siRNA *MAPK*.

Primary SFs were cultured from WT and *M_2_* mutant mice to measure cell proliferation by 5-bromo-2-deoxyuridine (BrdU) assay for up to 96 hours. The *M_2_* mutant SF cell proliferation was significantly reduced with time as compared with WT SFs (Fig. 4A; *P*=0.00004). In addition, the cell proliferation marker Ki67 was used to confirm the SF cell proliferation in cellular and protein level. A lower level of Ki67 expression was found in *M_2_* mutant SFs than in WT SFs (Fig. 4B; *n*=3).

### The effect of *M_2_* siRNA knockdown on SF cell proliferation

The cell growth pattern of *M_2_* (target gene)- and *MAPK1* (positive control)-transfected RNAi WT mouse primary SFs was monitored via xCELLigence cell impedance assay. There was a significant reduction in SF cell growth (measured in terms of cell index) observed after 64 hours of seeding (Fig. 4C) in *M_2_*-transfected mice compared with controls. Doubling time for the various types of transfected mouse SFs in the log phase of growth is also illustrated in Fig. 4C. It was shown that both the untreated and negative controls have a lower/shorter doubling time as opposed to *MAPK1*- and *M_2_*-silenced cells (a shorter doubling time equates to faster growth). It is expected that knockdown of the *MAPK1* gene, whose functions include an involvement in development and cell proliferation, will result in a slowdown in cell proliferation. An increase in doubling time was observed for *M_2_*-knockdown mouse SFs, which could be an indication that M_2_ might directly or indirectly decrease cell proliferation ability via interacting with other genes.

siRNA knockdown was performed over a period of 5 days on the mouse primary SFs and efficiency was validated using qRT-PCR to confirm an inhibition of at least 60%. Fig. 4D shows the gene expression profile of the target genes of mice after they were treated with their respective siRNAs. Both *M_2_* and *MAPK* (Fig. 4D) transfection showed a significant knockdown of the respective mRNA expression by day 3 and the levels were decreased further by day 5 (data not shown).

Immunohistochemical localization of M_2_ and MAPK in primary SFs was carried out at 72 hours after silencing. Positive immunostaining was observed for M_2_ in the untreated mouse SFs (Fig. 4E). In untreated SFs, M_2_ was mainly localized to the cell membrane and the cytoplasm (Fig. 4E), but expression was significantly reduced in the *M_2_*-silenced cells as observed by immunostaining and western blot (Fig. 4E). No immunostaining was observed in the negative controls and MAPK served as a positive control (Fig. 4F). β-tubulin was used as a loading control for protein expression. This result confirms that M_2_ was successfully knocked down in mouse SFs after 72 hours, which reduced the relative abundance of M_2_ in the cytoplasm and cell membrane.

### Changes in *M_2_* mRNA levels upon stimulation with muscarinic antagonists

**Fig. 5.**
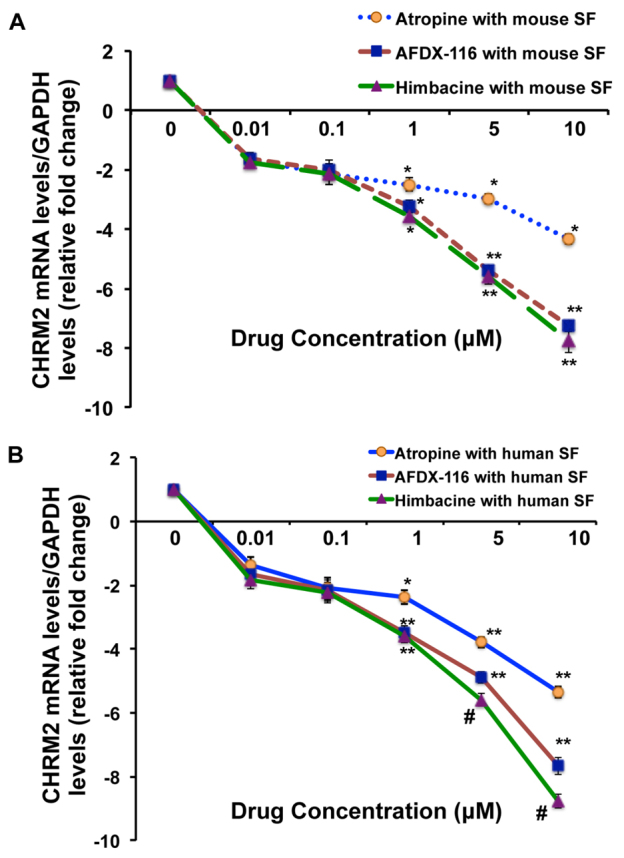
**Effect of muscarinic antagonists on SFs.** Passage 2 cultured human (A) and mouse (B) SFs were treated with atropine, AFDX-116, himbacine (at baseline, 0.1, 1, 10 and 100 μM) for 5 days. Following 5 days of treatment, the total RNA was extracted from these cells and *M_2_* (*Chrm2)* transcript levels were quantified via qRT-PCR analysis. Values were normalized against control values. The relative *M_2_* mRNA levels were downregulated by the three antagonists in a concentration-dependent manner in human and mouse SFs. Moreover, *M_2_* transcript level was most significantly reduced with himbacine treatment in human SFs (*P*<0.0001). The values represent means of five independent samples and error bars represent s.d.; *n*=5, *P<0.05, **P<0.01.

After treatment of primary SFs with varying concentrations of himbacine and 11-({2-[(diethylamino)methyl]-1-piperidinyl}acetyl)-5,11-dihydro-6H-pyrido(2,3-b)(1,4)benzodiazepin-6-one (AFDX-116; more selective to M_2_ receptors than is himbacine), and atropine, *M_2_* transcript levels were significantly reduced with all three drugs in a dose-dependent manner in both mouse (Fig. 5A) and human (Fig. 5B) SFs. This effect was more significant with himbacine in human SFs (*P*<0.00001) than with mouse SFs.

### M_2_ blockers retard myopia progression

**Fig. 6.**
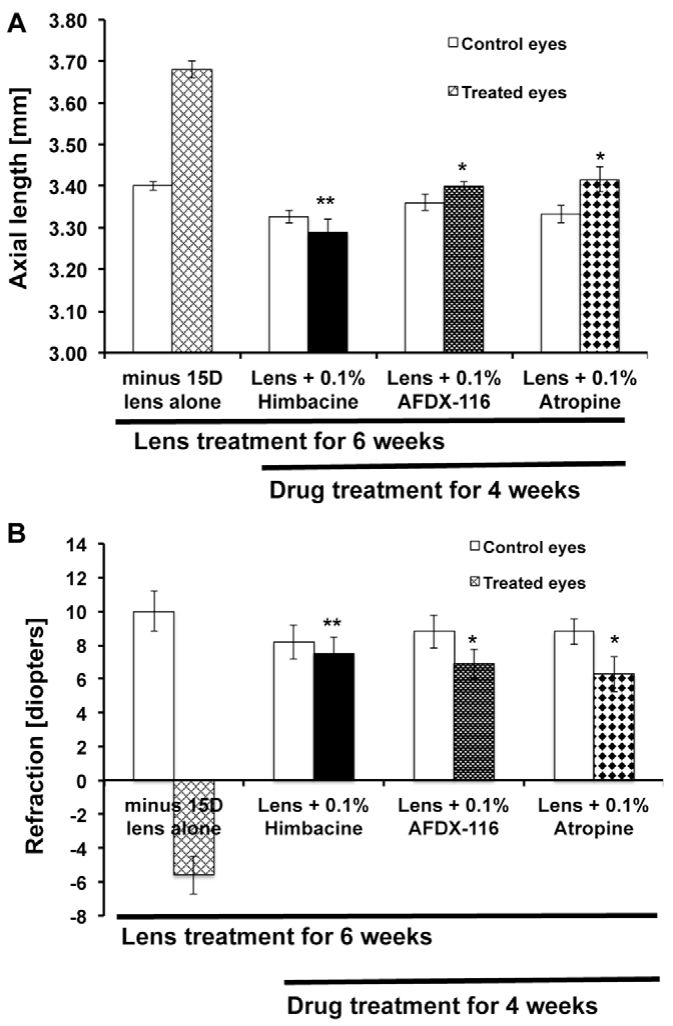
**WT mouse eyes were treated with different muscarinic antagonists and myopia progression was studied.** Mouse eyes treated with a −15D spectacle lens, lens with 0.1% himbacine, lens with 0.1% AFDX-116, and lens with 0.1% atropine for 2 weeks and 4 weeks (*n*=50 mice in each group). Right eyes were consistently used as the experimental eye and left eyes served as contralateral control. (A) Axial length was measured at 2 weeks and 4 weeks after treatment. The axial length was significantly reduced in the drug-treated eyes as compared with control and lens-treated eyes. All the drugs have significantly reduced the progression of myopia in mice (*P<0.01); however, himbacine showed the most significant amount of reduction in the mouse myopia progression (**P<0.0001). (B) An automated infrared photorefractor was used to perform refractive error measurements. The refractive error was shifted from myopic to hyperopic after receiving the drugs. Data are represented as mean ± s.d.; *P<0.01 and **P<0.0001.

In the present study, we determined whether M_2_-receptor-selective antagonists would be effective in reducing myopia progression in the mouse eye. Fig. 6A shows that axial length was significantly reduced in drug-treated (i.e. atropine, AFDX-116 and himbacine) eyes as compared with minus-lens-treated eyes (*n*=50 in each group). All three drugs showed a significant (*P*<0.01) reduction in mouse myopia progression; however, himbacine showed the most significant amount of reduction in the mouse myopia progression compared with the other drugs (*P*<0.0001). The refractive error was shifted from myopic to hyperopic after antagonist treatment (Fig. 6B). Himbacine and AFDX-116 are M_2_- and M_4_-receptor-specific antagonists with high affinity to M_2_. This result supports the crucial role of M_2_ in myopia. The fact that atropine had an effect that was similar to that of himbacine and AFDX-116 shows that atropine is acting directly on M_2_.

### Control of collagen gene expression with muscarinic antagonists

**Fig. 7.**
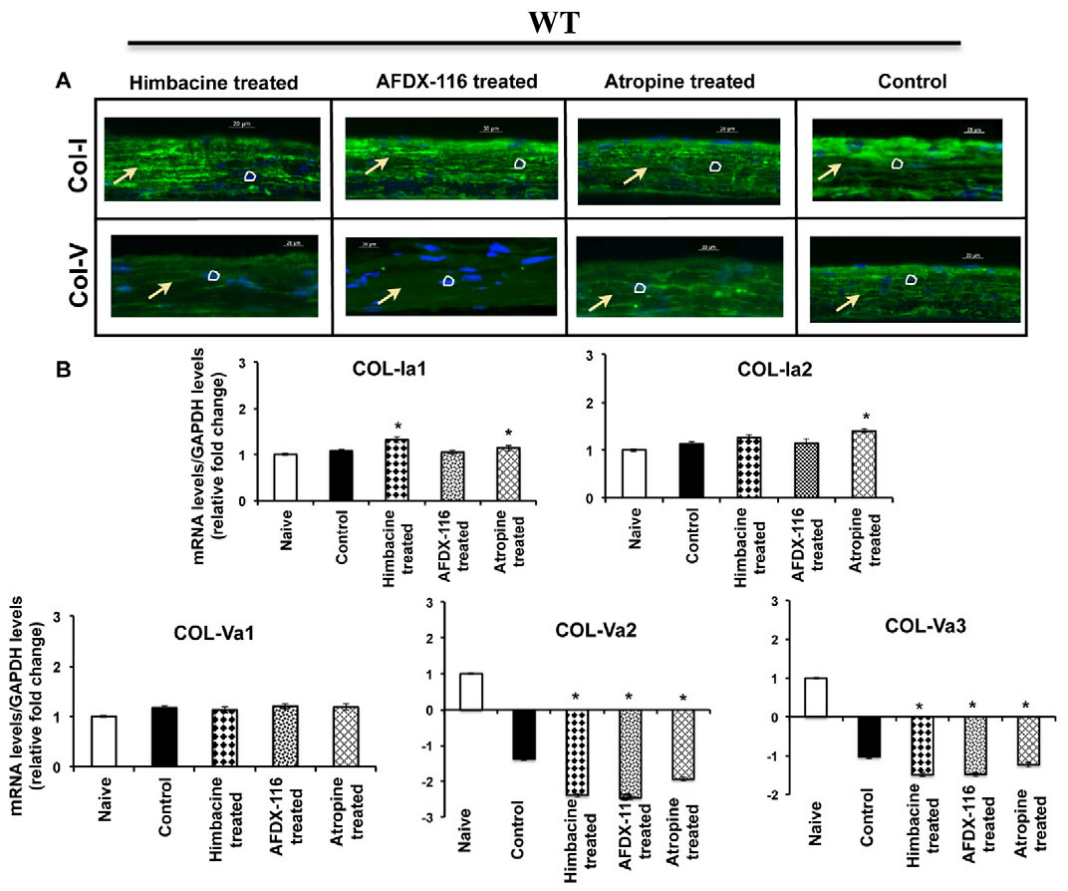
**Expression of collagen genes in sclera treated with muscarinic receptor antagonists.** (A) Type I collagen expression was increased in himbacine-, AFDX-116- and atropine-treated sclera compared with control sclera. This was opposite for the type V collagen expression. Arrows indicate sclera and circles represent nucleus. Scale bars: 20 μm. (B) The mRNA level of collagen 1a1, 1a2, 5a1, 5a2 and 5a3 in recovery sclera was compared with WT naive sclera after normalization with *GAPDH* reference gene. Collagen 1a1 and 1a2 were upregulated in the control and recovery sclera. However, collagen 5a2 and 5a3 were downregulated and no significant difference was determined in collagen 5a1. Data are represented as mean ± s.d.; *P<0.05.

The mRNA expression of collagen genes was investigated in myopic sclera with and without muscarinic-receptor antagonists to confirm the scleral remodeling process in mouse myopic and drug-treated sclera. The cellular level of *COL-1* expression in antagonist-treated sclera was almost close to control sclera; however, the expression of collagen type V was significantly low in the treated sclera compared with control sclera (Fig. 7A; *n*=3 independent samples). The transcript level of *COL-1A1* and *COL-1* was slightly upregulated in WT drug-treated sclera (recovery sclera) as compared with naive and control sclera (Fig. 7B; *n*=3 independent samples; *P*<0.00001), and this was statistically significant for M_2_/M_4_-specific himbacine-treated sclera. However, the mRNA expression of *COL-5A2* and *COL-5A3* was significantly downregulated in the recovery sclera and no significant difference was found in the *COL-5A1* mRNA level.

### Remodeling process and extracellular matrix interaction in myopic sclera

Many studies have shown a change in the activity of collagen and collagen-degrading enzymes in the sclera of myopic eyes (Birk et al., 1990; Rada et al., 2000; Siegwart and Norton, 2001; Gentle et al., 2003; Jobling et al., 2004; McBrien et al., 2006; McBrien et al., 2009a). In addition to collagen genes, we also determined the expression of fibronectin 1 (*FN-1*) and matrix metalloproteinase-14 (*MMP-14*) in WT and *M_2_* mutant minus-lens-induced myopic sclera with and without muscarinic antagonists. We also studied the mRNA expression of extracellular matrix (ECM)-related genes to confirm the remodeling process and ECM interaction in myopic sclera. The transcript level of integrin-B1, glycosaminoglycan, *FN-1* and transforming growth factor-β1,2,3 (*TGFβ*) was significantly downregulated in WT myopic sclera as compared with naive, control, *M_2_*-mutant lens-induced and drug-treated sclera (supplementary material Fig. S3; *n*=3; *P*<0.00001). However, the mRNA expression of *MMP-2* and *MMP-14* was significantly upregulated in WT myopic sclera as compared with naive, control, *M_2_*-mutant lens-induced and recovery sclera (*n*=3; *P*<0.0001).

## DISCUSSION

This is the first *in vivo* study implicating a role of M_2_ in myopia development in a mouse model. The present study validates the functional role of M_2_ in a knockout mouse model and identifies an M_2_ muscarinic receptor (which is encoded by the *Chrm2* gene) as a key protein involved in the development of myopia. We show that mice lacking M_2_ have growth-related changes in the expression of ECM genes and are less susceptible to the induction of experimental myopia. Additionally, pharmacological blockade of M_2_ muscarinic receptors retarded myopia progression in WT mice. Our *in vitro* study of WT mouse SF cell proliferation showed a significant reduction in SF cell growth after 3 days of *M_2_* siRNA knockdown. *M_2_*-mutant mouse SF cell growth was also shown to be significantly slower than that of WT mice *in vivo*. These results strongly suggest that M_2_ could control SF cell proliferation and growth.

To date, atropine (Tong et al., 2009) and pirenzepine (Siatkowski et al., 2008) have been shown to retard the progression of myopia in children but, because the molecular basis of myopic progression has not been clear, it has not been feasible to develop specific targeted antagonists. Atropine works with an unknown non-specific target, so we are uncertain of the required duration of treatment and long-term side effects (Tong et al., 2009). Moreover, little is known about the receptors and/or proteins that are responsible for shortening axial length. A few studies have been reported that used muscarinic receptor antagonists of M_1_, M_2/4_, M_3_ and M_4_ to arrest myopia progression in animal models (Cottriall et al., 2001; Luft et al., 2003). Because of the lack of small-molecule ligands that can block specific muscarinic receptor subtypes with high selectivity, muscarinic receptor knockout mice as used here represent highly useful experimental tools (Wess et al., 2007). Results from the present study suggest that M_3_ had a similar change of axial growth and refractive error as M_2_. These results indicate that there is a relationship between M_2_ and M_3_ subtypes in myopia development. The distributions of muscarinic acetylcholine receptors within the ocular tissues are shown in supplementary material Table S1. The M_2_ and M_3_ subtypes predominate in sclera and retina, but their functions in eyes have not been explored in detail. Previous studies reported that both the M_2_ and M_3_ subtypes could induce smooth muscle contractions (Matsui et al., 2002; Unno et al., 2005) and neurotransmission (Gosens et al., 2006). This shows that the M_2_ and M_3_ subtypes might mediate the progression of myopia or scleral remodeling through similar ion channels or are probably activated either by second messengers or by direct receptor actions. This has to be further explored with selective blockers and double-knockout mice.

Anti-muscarinic drugs altered the expression of scleral collagen and other structural molecules in animal models of myopia (Lind et al., 1998; Cottriall et al., 2001; Luft et al., 2003; McBrien et al., 2006; Bitzer et al., 2006), establishing a link to scleral connective tissue in these pathways. However, even though muscarinic receptor subtypes might be involved in scleral remodeling, the downstream signaling pathways have not been identified. In this study, we show that the functional loss of M_2_ reduces axial elongation and increases scleral thickness through control of collagen production, and that pharmacological blockade of M_2_ receptors in WT mice reduces the progression of myopia. This is the first study to show that reduced type I collagen and increased type V collagen controls scleral tissue loss in the development of myopia. This suggests that the newly synthesized fibrils in myopic sclera contain more type V collagen, a finding that is supported by previous studies conducted in humans and tree shrews (Birk et al., 1990; McBrien and Gentle, 2001). Our findings speculate that the loss or alteration of M_2_ in sclera might change the magnitude of collagen production and ECM interaction in myopic eyes. TGFβ was shown to be the main regulator for collagen production in sclera via fibroblasts (Jobling et al., 2004). The present study results also show that *TGFβ* transcripts modulate in myopia, which might link to the alterations of ECM production and collagen-degrading enzymes in the sclera of eyes developing myopia. Therefore, the present study shows that sclera is a potential site where muscarinic antagonists can target in order to slow down myopia progression. Taken together, either M_2_ alone or in interaction with collagen and other molecules plays a central role the development and/or progression of myopia (supplementary material Fig. S4).

It is important to identify young children who are at high risk of developing myopia (with parental myopia, increased near work, lack of outdoor activity) for gene testing that will propose target drug intervention to decrease the progression of myopia. As mentioned above, previous studies have shown that atropine has been proven clinically to arrest myopia progression in children (Tan et al., 2005; Chua et al., 2006; Tong et al., 2009; Ganesan and Wildsoet, 2010). However, atropine shows many unwanted adverse effects, such as glare, photophobia and blurring of near vision. This is due to the non-specificity of the drug or due to the mydriasis effect of the drug (Tong et al., 2009). To overcome these side effects, it is essential to design a specific M_2_ blocker to retard myopia progression. This study shows that atropine and other muscarinic antagonists are likely to exert their beneficial effects via blockade of the M_2_ muscarinic receptor subtype. Therefore, the present study indicates that an antagonist that is highly selective for M_2_ receptors might become useful for the treatment of myopia.

M_2_ is highly conserved in human and mouse, with 89% nucleotide and 96% amino acid similarity. Several myopia-related genes have been reported recently from GWAS looking at single nucleotide polymorphisms (SNPs). Three genes for myopia, in 15p14 (*GJD2*, *ACTC1*) and 15q25 (*RASGRF1*), were identified to be susceptible loci in European Caucasians (Hysi et al., 2010; Solouki et al., 2010). In another GWAS on Asian cohorts, the gene *CTNND2* was found to be highly associated with high myopia (Li et al., 2010). In these studies, functionality of the genes was not well characterized, although RASGRF1 had been reported previously to play a role in retinal function and morphogenesis (Fernández-Medarde et al., 2009). In comparison, we have demonstrated strongly the role of M_2_ in experimental myopia. Genomic conservation strongly suggests important functionality and, given the functional role of M_2_ in the mouse model, it warrants further investigation in human studies, especially its therapeutic potential.

### Limitations

In the present study, *M_2_* muscarinic cholinergic receptor knockout mice were used to determine the functional role of M_2_ in myopia. In these mice, the *M_2_* gene is disrupted in all cells of the body. In future studies, we are planning to develop tissue-specific M_2_ receptor knockout mice to identify the target tissues involved in M_2_-receptor-dependent myopia.

### Conclusion

In summary, our study shows that M_2_ mediates myopia, and blockers of this receptor are potential drugs for the reduction of myopia progression. These results suggest a major role of M_2_ in the scleral growth-related changes during myopia development. Our drug study results imply that an antagonist that is highly selective for M_2_ receptors (which is not currently available) might become useful for the treatment of myopia.

## MATERIALS AND METHODS

### *M_2_* mutant mice

Heterozygous *M_1_*–*M_5_* mutant mice were backcrossed for 12 generations to C57BL/6NTAC to achieve genomic homogeneity of 99.95% (Wess et al., 2007) then cross-bred in the animal holding unit of SingHealth Experimental Medical Center and genotyped. Naive control animals were housed in groups of six, whereas experimental animals were housed individually after the age of 28 days at 25°C on 12:12 hours of light:darkness, with mouse pellets and water available *ad libitum*. Approval was obtained from the SingHealth Institutional Animal Care and Use Committee (IACUC; AAALAC accredited) and all procedures performed in this study complied with the Association of Research in Vision and Ophthalmology (ARVO) Statement for the Use of Animals in Ophthalmology and Vision Research.

### Human tissues

Human scleral tissues (*n*=10) harvested within 24 hours from normal cadaver eyes (age range 35–70 years) obtained at autopsy were provided by the Singapore Eye Bank. The protocol was approved by the Institutional Review Board of the Singapore Eye Research Institute, Singapore. All study procedures were performed as part of standard clinical care and complied with the tenets of the Declaration of Helsinki regarding human research. Because all the procedures performed were essential for standard clinical care of these patients, written consent was not required, but consent was obtained by assent. The patients’ next of kin were aware of the privacy policy of the hospital, which states that information released for publication would not include patient identifiers.

### Murine myopia model

A −15 diopter spectacle lens [PMMA Contact Lens (Lenspec, Singapore) in Grey Tint, 8.5 mm diameter, 8 mm base curve, refractive index: 1.43, axial thickness: 0.5 mm] was placed over the right eye on day 10 by gluing to an annulus of velcro, and then attaching to a matching piece of velcro that had been previously sutured to the skin around the eye. The spectacle lenses were cleaned daily in dim light and left eyes were uncovered and served as controls. All optical interventions were removed on postnatal day 52 (Barathi et al., 2008; Barathi and Beuerman, 2011).

### Ocular biometry assessment

Each eye was refracted every week to measure the refractive error using an automated infrared photorefractor (Image Source, Kirkland, WA). By using OLCI, AC Master (Carl-Zeiss Meditec, Oberkochen, Germany), the biometry of the eye was measured *in vivo* (Barathi et al., 2009b; Schaeffel et al., 2004) at 24 days, 38 days and 52 days after induction of myopia. This method has been demonstrated to provide improved resolution and reproducibility, and allowed serial monitoring of axial length of the eyeball in various stages of myopic induction. Differences of refractive power and axial length between eyes were calculated. Differences of refractive power, axial length, corneal thickness, anterior chamber depth, lens thickness and vitreous chamber depth between treated and control eyes were calculated.

### Immunohistochemistry

The whole mouse eye (6 weeks lens-treated and control eyes; *n*=6) were embedded in OCT (Optimal Cutting Temperature) compound (Ted Pella, Inc. and PELCO International, Redding, CA) at −20°C for 1 hour. Tissue blocks were sectioned with a cryostat (HYRAX C 50, Carl Zeiss Microimaging GmbH, Germany) at 5 μm thicknesses and collected on a clean Polysine™ microscope glass slides (Gerhard Menzel, Thermo Fisher Scientific, Newington, CT). Sections were air dried at room temperature (RT) for 1 hour and fixed with 4% paraformaldehyde for 10 minutes. After washing three times with 1× phosphate buffered saline (PBS) for 5 minutes, 4% goat serum diluted with 1× PBS was added as a blocking buffer. The slides were incubated for 1 hour at RT in a humid chamber. After rinsing with 1× PBS, a specific primary antibody against collagen type I (COL-I; Abcam, Cambridge, UK), collagen type V (COL-V; Abcam, Cambridge, UK), muscarinic cholinergic receptor-2 (M_2_), fibronectin-1, matrix metalloproteinase-14 (MMP-14; Chemicon, Temecula, CA) and Ki-67 cell proliferation marker (Santa Cruz Biotechnology, Inc., Santa Cruz, CA) diluted (1:100) with 2% goat serum was added and incubated overnight at 4°C. After washing three times with 1× PBS for 10 minutes each, fluorescein-labeled goat anti-rabbit secondary antibody (1:200; Chemicon) was applied and incubated for 90 minutes at RT. After washing and air drying, slides were mounted with antifade medium containing DAPI (4,6-diamidino-2-phenylindole; Vectashield; Vector Laboratories, Burlingame, CA) to visualize the cell nuclei. Sections incubated with 2% goat serum instead of primary antibody served as negative control. Experiments were repeated in triplicates from three independent batches of samples.

Eye sections from WT and *M_2_* mutant mice (*Chrm2*^+/+^, *Chrm2*^+/−^, *Chrm2*^−/−^; *n*=6 eyes from each strain) were prepared as above for the staining of sections with antibodies specific for M_1_–M_5_. Endogenous peroxidase activity was used to block the non-specific binding sites. The slides were subsequently treated with appropriate secondary antibodies conjugated to biotin, then developed utilizing avidin-conjugated horseradish peroxidase (HRP) with diaminobenzidine (DAB) as substrate (Vectastain ABC Kit from Vector Labs, Burlingame, CA). Following development, the slides were counterstained for contrast, and mounted under coverslips with permount. After adequate drying, these slides were then ready for imaging. The sections stained with IgG without the primary antibody were used as a control.

### Primary cell culture and drug treatment

Human scleral tissues (*n*=10) and 8-week-old mouse sclera (*n*=100 eyes, 20 sclera/batch) from post-mortem eyes were obtained. The whole sclera was dissected very carefully and washed with cold PBS three times. Fibrous sclera was placed in a 60 mm culture dish with Dulbecco’s modified Eagle’s medium (DMEM; Gibco) supplemented with penicillin, streptomycin and amphotericin B and 10% fetal bovine serum (FBS; Gibco). Cultured cells were incubated at 37°C, 5% CO_2_ and allowed to reach 80% confluence. Cells were passaged sequentially by exposing cells to 0.25% trypsin/0.5 mM EDTA at 37°C for 5 minutes. The culture conditions were as previously described (Barathi et al., 2009a).

### Cell proliferation assay

SFs were passaged from *M_2_*^−/−^ and *M_2_*^+/+^ mouse scleral tissues and used between passages 1 and 2. Cell proliferation was assessed by measuring BrdU incorporation during DNA synthesis in proliferating cells (Oncogene, Cambridge, MA). For the cell proliferation assay, 100 μl of passaged SFs (1×105 cells/ml) were seeded into 96-well plates containing DMEM with 10% FBS. The method was followed as described previously (Barathi et al., 2009a). The color reaction was stopped, and the optical density was determined using a Spectrafluor Plus microplate reader (TECAN, Durham, NC), set to 450–595 nm.

### *M_2_* siRNA knockdown

Mouse oligonucleotides that were specific for *M_2_* siRNA were purchased from Qiagen (Qiagen, Hilden, Germany). A positive control siRNA that targets the protein kinase *MAPK1* was also used. In addition, a negative control siRNA, which is not homologous to mammalian genes, was used to control for non-specific silencing effects. The target sequences for the *M_2_* and *MAPK* oligonucleotides can be found in supplementary material Table S2. The siRNA transfection of the cells was performed with the HiPerfect Transfection Reagent (Qiagen) in accordance with the instructions provided by the manufacturer. Analysis of the silencing effects was determined by immunocytochemistry, qRT-PCR and western blotting, and the level of knockdown achieved was calculated according to the manufacturer’s instructions (Qiagen).

### Cell culture and transfection

B6 mouse SFs of passage 4 were used. 3.5×10^4^ mouse SF cells were plated in a 12-well plate and maintained in DMEM supplemented with 10% FBS and 1% penicillin and 1% streptomycin at 37°C 5% CO_2_ prior to transfection. All siRNA used were purchased from Qiagen. Transfection was performed following the manufacturer’s instructions. Briefly, 30 nM of various siRNA – *MAPK1* siRNA (positive control GenBank accession number NM_011949), scrambled siRNA negative control and *M_2_* siRNA (NM_203491) – were diluted in basal DMEM incubated with HiPerfect transfecting reagent at RT for 10 minutes to allow the formation of transfection complexes. The complexes were added drop-wise onto the cells and then incubated to monitor the gene silencing for the next 96 hours. Finally, the gene knockdown efficiency was determined using qRT-PCR.

### Monitoring cell growth in real time

Roche xCELLigence system RTCA SP (Roche Applied Science, IN) was used for the monitoring of cell proliferation in real time. Cells were seeded in microtiter plates containing microelectronic sensors (96X E-Plate). The interaction of cells with the electronic biosensors generates a cell-electrode impedance response that is expressed as cell index, which allows for cell numbers to be detected. Cells that had previously been transfected were trypsinized and counted. 3500 mouse SF cells transfected with various siRNA were seeded in 100 μl of media in duplicates in a 96X E-Plate, and proliferation was monitored in real time. Cell-sensor impedance was measured every 5 minutes for the first 2 hours, every 15 minutes for the next 6 hours and once every hour for the rest of the experiment.

### *In vitro* drug treatment

All cells used in the experiments were from passages 2–4. Passaged cells were plated at a concentration of 2×10^5^ cells/ml into six-well plates containing DMEM with 10% FBS. The cells were seen to attach to the bottom of the culture wells after 4 hours. Mouse and human SFs were treated with atropine, AFDX-116 or himbacine (0.1, 1, 10 and 100 μM) for 5 days. Drugs were freshly prepared in DMEM with 10% FBS. Old media was removed from wells and 1 ml of drug was added with fresh full medium and incubated at 37°C. Drugs were replaced daily for the next 5 days to avoid drug degradation. The drugs/media was removed and wells were washed twice with 1× PBS. Cell lysate was collected for relative qRT-PCR at day 5.

### Immunocytochemistry

Mouse SFs were cultured on eight-chamber sterile slides before being transfected with siRNA. 72 hours following transfection, the cells were washed twice with 1× PBS (1st BASE) before being fixed in ice-cold methanol-acetone (1:1) for 10 minutes. They were then kept at −20°C. Before blocking was performed, the chamber slides were air dried in the fume hood for 10 minutes. Blocking was then carried out on the non-specific sites using 1% BSA (BSA; Sigma-Aldrich) for 15 minutes. After that, the cells were incubated overnight at 4°C with the following primary antibodies diluted in 1× PBS (1st BASE): anti-M_2_ (Abcam) and -p44/42 MAP kinase (ERK1/2) rabbit polyclonal antibody (#06-182; Upstate Biotechnology, MA). The dilution factor was 1:100. Washing was done on the cells three times using 1× PBS with a 5-minute interval in between. The cells were then incubated with horseradish peroxidase (HRP)-conjugated secondary antibodies (#185841; Pierce Biotechnology, Rockford, IL) diluted in 1% PBS and BSA (Sigma-Aldrich) at a dilution of 1:200 in the dark for 1 hour at RT. In the dark, the cells were washed three times in PBS with an interval of 5 minutes in between before being air dried. An antifade medium containing DAPI (Vectashield; Vector Laboratories, Burlingame, CA) was then added drop-wise to the slides. Observation of the slides was carried out using a fluorescence microscope (Axiophan 2; Carl Zeiss Meditec GmbH, Oberkochen, Germany) and images were saved.

### Protein extraction

Mouse SF cells were harvested with 0.25% trypsin/0.5 mM EDTA (Sigma-Aldrich) diluted in PBS (1st BASE). They were then centrifuged at 14,000 ***g*** for 10 minutes at 4°C before the trypsin was removed. 100–200 μl of ice-cold radioimmunoprecipitation assay (RIPA; Santa Cruz Biotechnology, CA) lysis buffer with phosphatase inhibitor was then added and the cells were left to incubate for 30 minutes. The cells then underwent sonication in three pulses, each for 5 seconds with a 1-minute break in between. The homogenized cells were again centrifuged at 14,000 ***g*** for 10 minutes at 4°C, after which the supernatant was removed and used as total cell lysates. All protein samples were kept at −20°C.

### Western blot

Mouse scleral proteins (*Chrm2*^+/+^, *Chrm2*^+/−^, *Chrm2*^−/−^, 6 weeks lens-treated and control WT eyes; *n*=6 eyes from each strain) in the supernatant were separated by SDS-PAGE, transferred to nitrocellulose membranes, blocked in 5% BSA in TBST [10 mM Tris-HCl (pH 8.0), 150 mM NaCl and 0.0.05% Tween-20] for 2 hours at RT, and incubated with the same anti-M_2_ antibody described above at a dilution of 1:1000 and anti β-tubulin antibody used as a loading control, for 1 hour at RT. The membranes were washed three times in TBST and incubated with HRP-conjugated secondary antibody (Chemicon International) at a dilution of 1:2500 for 1 hour at RT. Immunoreactive bands were visualized using the enhanced chemiluminescence method (GE Healthcare, Buckinghamshire, UK). The membrane was wrapped in plastic and placed against an X-ray film to expose for an appropriate length of time (30 seconds to 5 minutes).

### RNA preparation, RT-PCR and qRT-PCR

Total RNA was isolated from six mouse sclera of each strain (*Chrm2*^+/+^, *Chrm2*^+/−^, *Chrm2*^−/−^, 6 weeks lens-treated and control WT eyes; *n*=6 eyes), and from human and mouse SF cell lysates with siRNA transfection and after drug treatment using the MELT™ Total Nucleic Acid Isolation System (Ambion Inc., Austin, TX) according to the manufacturer’s instructions. RNA concentration and quality were assessed by absorbance at 260 nm and the absorbance ratio of 260/280, respectively, using Nanodrop^®^ ND-1000 Spectrophotometer (Nanodrop Technologies, Wilmington, DE).

cDNAs were synthesized using iScriptTM select cDNA synthesis kit (Bio-Rad Laboratories, CA). cDNA concentration and quality were assessed by absorbance at 260 nm and the absorbance ratio of 260/280, respectively, using Nanodrop^®^ ND-1000 Spectrophotometer. The cDNAs from each sample were amplified for muscarinic receptors via reverse-transcriptase PCR (RT-PCR) and transcript level was quantified by qRT-PCR as previously described (Barathi et al., 2009a). The experiments were repeated with three independent batches of samples. The primer sequences of all genes are presented in supplementary material Table S2.

### *In vivo* drug treatment

The effects of treatment with muscarinic antagonists were examined in B6 WT mice after induction of myopia with a −15D spectacle lens. There were three different groups: the first group (*n*=50) received a daily 1 drop of 0.1% atropine sulfate, the second group received a daily 1 drop of 0.1% himbacine and the third group received a daily 1 drop of 0.1% AFDX-116. Topical applications were administered to both eyes at the same time each day (∼9:00 am) commencing on the 24th day (2 weeks after spectacle lens treatment). A compatible drug level was determined prior to the *in vivo* use in a tissue culture study with mouse SFs. These concentrations (0.01%, 0.1%, 0.5% and 1%) were then tested *in vivo* in a small pilot study (data not shown). In this study, we are reporting results from the 0.1% drug treatment. The eyes were examined daily and no infections were found. This treatment schedule continued for 4 weeks starting on postnatal day 24 and continuing until postnatal day 52. All measurements were taken at postnatal day 52, the equivalent of 6 weeks of spectacle lens wear and 4 weeks of drug treatment.

### Data analysis for mouse model

Statistical comparisons between experimental groups were conducted using Student’s *t*-test or one-way ANOVA (Statistica 6.0, SPSS, Chicago, IL), followed by Tukey post-hoc test. A significance level of *P*<0.05 was used. Data are presented as means ± standard deviation.

## Supplementary Material

Supplementary Material
