# Supplementary material for: Muscarinic cholinergic receptor (M2) plays a crucial role in the development of myopia in mice
Source: Dis Model Mech. 2013 May 2;6(5):1146–58. doi: 10.1242/dmm.010967 (PMC3759334; doi:10.1242/dmm.010967)
Supplement: Supplementary Material [file supp_6_5_1146__index.html]

Muscarinic cholinergic receptor (M2) plays a crucial role in the development of myopia in mice — Muscarinic cholinergic receptor (M2) plays a crucial role in the development of myopia in mice — Supplementary Material 

# Muscarinic cholinergic receptor (M2) plays a crucial role in the development of myopia in mice

## 

**Files in this Data Supplement:**

- **Supplementary Material PDF**
