# Supplementary material for: Muscarinic cholinergic receptor (M2) plays a crucial role in the development of myopia in mice
Source: Dis Model Mech. 2013 May 2;6(5):1146–58. doi: 10.1242/dmm.010967 (PMC3759334; doi:10.1242/dmm.010967)
Supplement: Supplementary Material [file supp_010967_DMM010967.pdf]

Supplementary Materials

Supplementary Figure 1: Myopia induction was performed using uniocular -10 and -15 diopter negative lenses in wild-type and homozygous CHRM2-deleted mice, and results at 2, 4 and 6 weeks shown. The lens was applied at post-natal day 10 (before eye opening). The ocular biometry measurements were measured using the OLCI-AcMaster (in vivo: accuracy ± 10 microns) and refraction (diopters) was measured by automated infrared photorefractor at 2 weeks, 4 weeks and 6 weeks after induction of myopia. 2A: The refraction (diopter) was plotted against a lens wearing period (weeks). The mice ocular biometry measurements (mm) were plotted against the lens wearing period (weeks). The corneal thickness (2C) and anterior chamber depth (2D) was not significantly different when comparing the lens treated eyes against contra-lateral control eyes in both strains. The axial length (2B), lens thickness (2E) and vitreous chamber depth (2F) were significant after 4 weeks and 6 weeks of induction of experimental myopia in WT when compared to *CHRM2*-mutant mice (n=50, p <0.01 and n = 50, p = 0.08 respectively). However when comparing with the induction period, the WT mice eyes were more significant at 6 weeks of minus lens wearing (n = 50, p<0.001). Spectacle lens-induced myopia caused elongation of the globe and reduced hyperopia in WT mice. *CHRM2*-mutant mice were immune to myopia induction. Data was represented as mean ± S.D, * p<0.05, ** p<0.01 and *** p<0.001.

Supplementary Figure 2: Immunohistochemistry, Western blot and Reverse Transcriptase Polymerase Chain Reaction (RT-PCR) studies with *CHRM1-5* mice.

A. To determine M1-M5 expression in the mouse eye, whole eye sections (5 microns) were subsequently treated with appropriate secondary antibodies conjugated to biotin, then developed utilizing avidin-conjugated horseradish peroxidase (HRP) with

diaminobenzidine (DAB) as substrate. Following development, the slides were counterstained for contrast, and mounted under cover slips with permount. After adequate drying, these slides were then ready for imaging. Positive immunostaining of M1-M5 was identified in the *CHRM2 WT (*+/+) and *CHRM2* heterozygote (+/-) mouse retina pigment epithelium (RPE), choroid and sclera, and not found in the homozygous *CHRM2*-mutant (-/-) mouse. Arrows indicate sclera Scale bar = 50 m. Magnification at 400X.

B. Western blotting studies confirm the presence of M1-M5 protein in +/+ and +/- scleral tissue and its absence in *CHRM2*$^{-/-}$ mice. Beta-tubulin was used as a loading control.

C. RT-PCR shows the expression of all five *CHRMs* in the sclera of *CHRM2* +/+ and +/- mice, and the absence of *CHRM2* transcription in the *CHRM2* -/- mouse. GAPDH was used as a reference gene. L- DNA ladder; M_1*-CHRM1;* M_2*-CHRM2;* M_3*-CHRM3;* M_4*-CHRM4;* M_5*-CHRM5.*

Supplementary Figure 3: Interaction of ECM genes in myopic and drug treated sclera.

The mRNA level of ECM genes in myopic and drug treated sclera was compared with WT naive sclera after normalized with GAPDH reference gene. Integrin- 1, Fn-1, TGF- 1, 2, 3 was down regulated in the WT myopic sclera and MMP-2, MMP-14 was up regulated. This was reversed in the atropine, AFDX-116 and himabcine treated myopic sclera. Data was represented as mean ± S.D, * represents significance level p<0.01 and ** represents significance level p<0.0001.

Supplementary Figure 4: Schematic diagram shows interaction of collagen, ECM molecules and M2, and its downstream signaling pathway. Muscarinic receptors have been classified into two groups based on their functional coupling. The first group

consists of M1, M3 and M5 subtypes, which are coupled to G q/11 protein that activates phospholipase C (PLC). This leads to the formation of inositol 1,4,5-triphosphate (IP3) and diacylglycerol (DAG) which in turn mobilizes Ca2+ and activates Protein Kinase C (PKC) respectively. The second group consists of M2 and M4 receptors involve the activation of inhibitory subunit of G protein (G i), which inhibits GTP and adenylyl cyclase, thereby reducing cyclic adenosine monophosphate (AMP) level. This suggests a possible interaction between CHRM and other GPCR molecules through endogenous ADCY. This pathway suggests that interaction of collagen, ECM molecules and CHRM2 could regulate ERK1/2 Mitogen-Activated Protein Kinase (MAPK) signaling pathway in scleral fibroblasts through GPCRs. Double-headed solid arrows represent reversible changes; single-headed solid arrows represent direct regulation; double-headed dotted arrow represents possible interaction.

Supplementary Figure 1

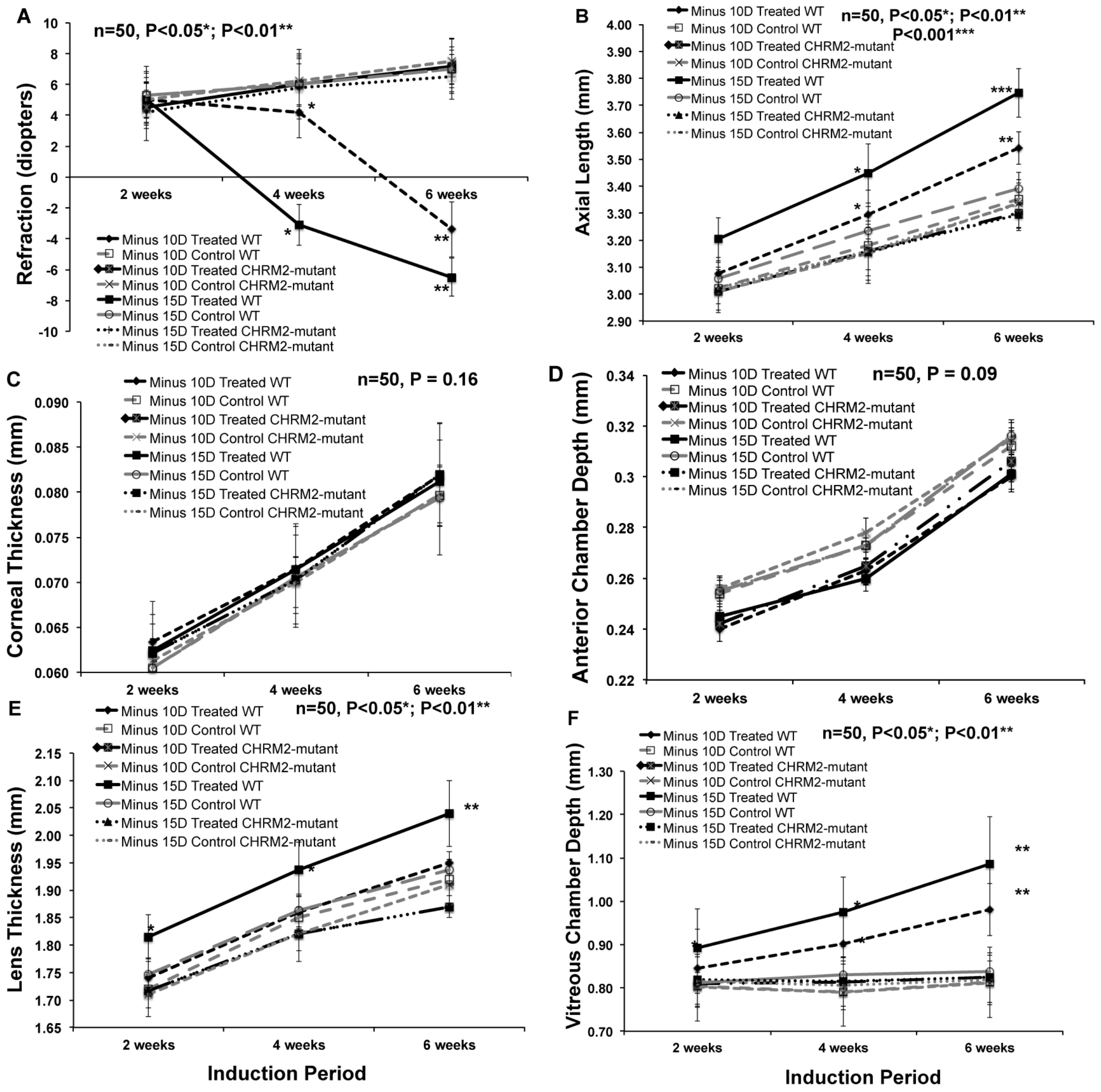

Supplementary Figure 2

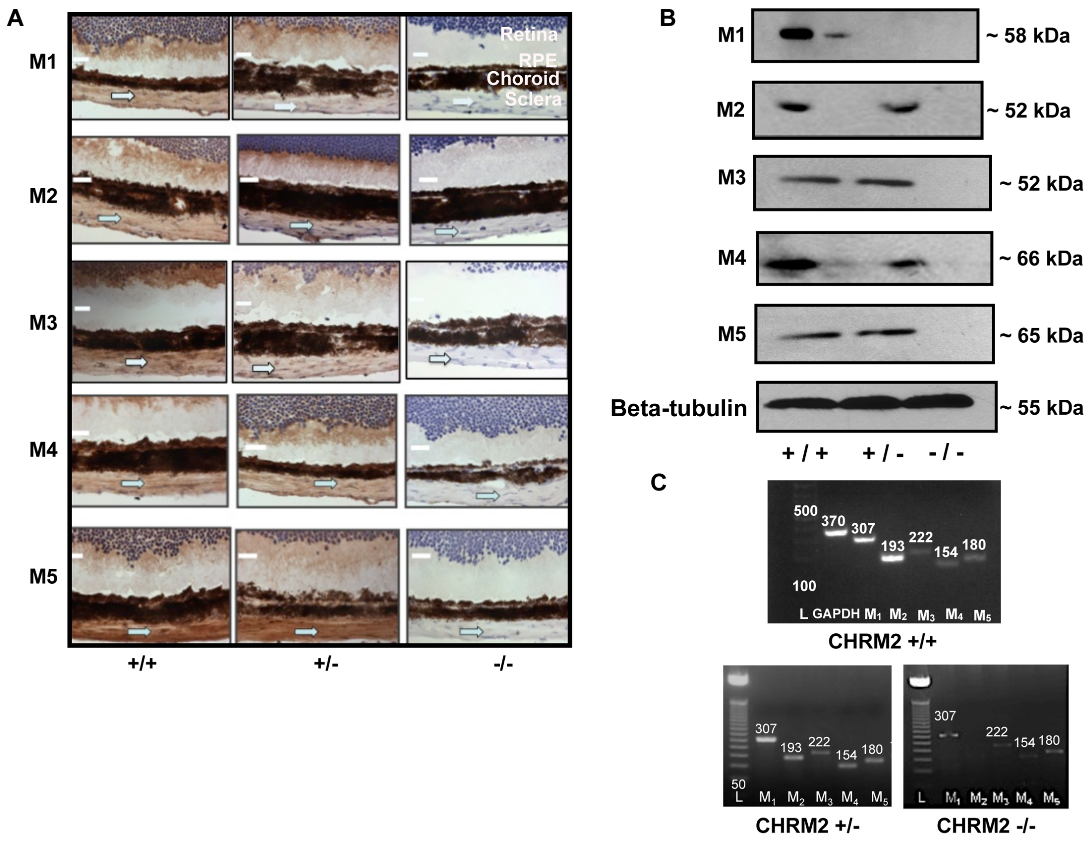

Supplementary Figure 3

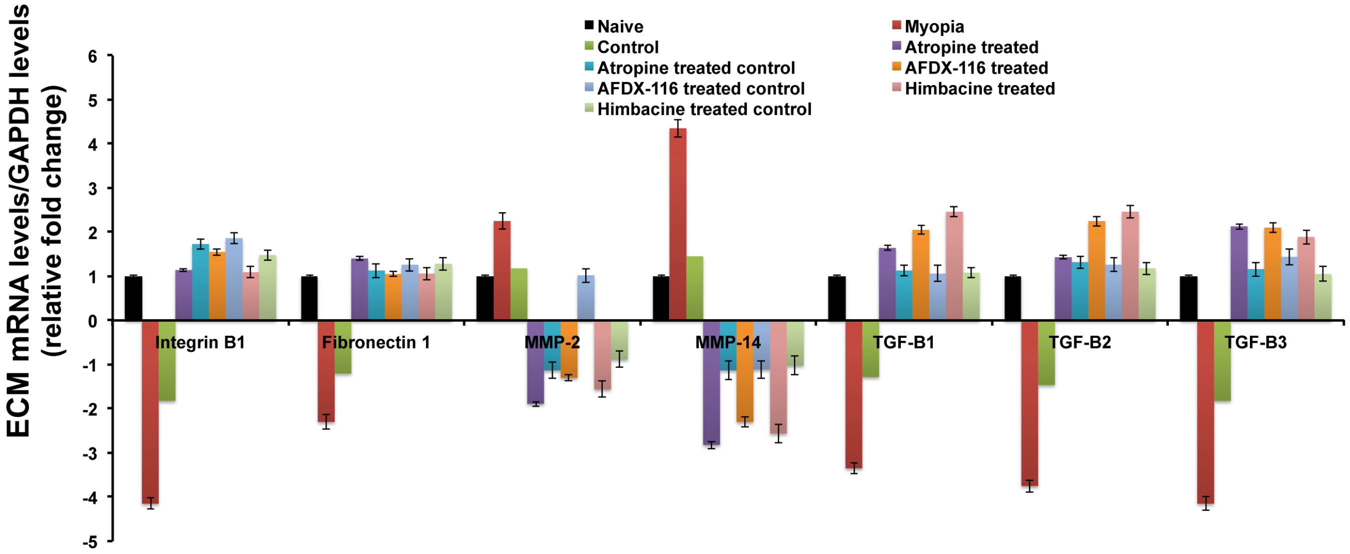

Supplementary Figure 4

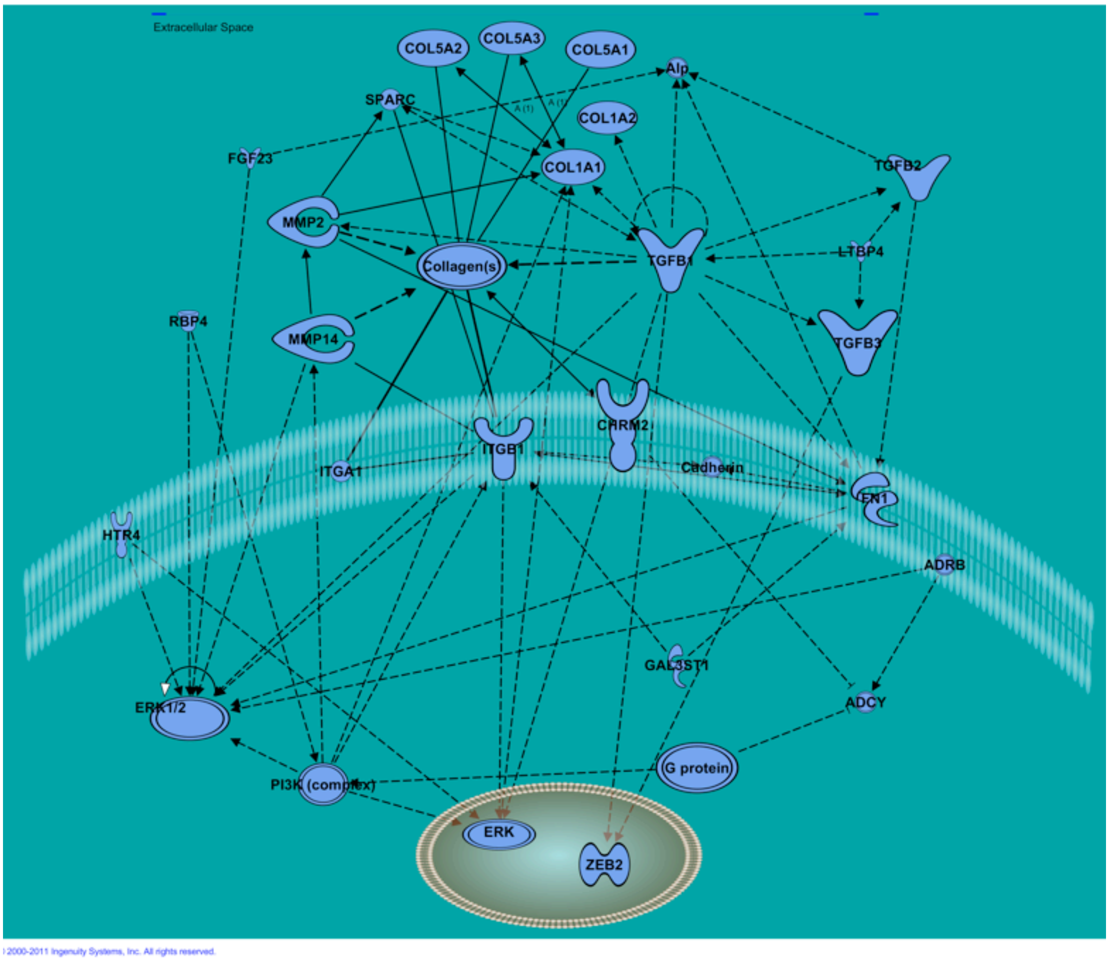

Supplementary Table 1: The distribution of muscarinic acetylcholine receptors within the ocular tissues in various species. *CHRM*1-5 is commonly referred as M1-5 respectively. The presence of muscarinic receptor subtype in the species is represented by ■.

**Supplementary Table 1. Muscarinic Acetylcholine Receptors in ocular tissues**								
**Species**	**Subtypes**					**Localization(s)**	**Remarks**	**Reference(s)**
	**M_1**	**M_2**	**M_3**	**M_4**	**M_5**			
Human	■	■	■	■	■	Sclera, retinal pigment epithelium and choriod	M_3 predomniates in retina, sclera,cornea, iris, ciliary body and lens	(Friedman et al., 1988; Qu et al., 2006; Barathi et al., 2009a)
Mouse	■	■	■	■	■	Retina, retinal pigment epithelium, choroids, ciliary bodies and sclera	M_1 & M_4: retina, choroid, ciliary body and sclera	(Barathi et al., 2009a)
Monkey		■	■	■		Conjunctiva, Meibomian glands, suprabasal layers of the skin epithelium	M_2-M_4: conjunctiva<br><br>M_3 - M_4: suprabasal layer of skin	(Liu et al., 2007)
Chick		■	■	■		Retina, retinal pigment epithelium, choroid and ciliary body		(Fischer et al., 1998)
Tree-shrew	■	■	■	■	■	Retina, choroid, sclera and iris/ciliary body	M_1 & M_4: retina, choroid, ciliary body and sclera, M_2: ciliary body<br>M_3 and M5: all	(McBrien et al., 2009b)
Guinea pig	■	■	■	■	■	Retina, choroid, sclera, and iris-ciliary body		(Liu et al., 2007)
Bluegill Fish	■	■			■	Retinal pigment epithelium		(Phatarpekar et al., 2005)

Supplementary Table 2. Gene accession number and qPCR primer sequences

Genes	Accession Number	Forward Primer	Reverse Primer
M1	NM_007698	5’ gac cct aca gac ccc tct cc 3’	5’ gca gcc agg tgg aat gaa 3’
M2	NM_203491	5’ ccc caa tac agt gtg gac aa 3’	5’ gca ggg ttg atg gtg cta tt 3’
M3	NM_033269	5’ ttc cca tca tga tac aca cca 3’	5’ aat gtc acg tgc ttg gtc ac 3’
M4	NM_007699	TaqMan Primers	
M5	NM_205783	5’ agg tga aaa tca tgc cct gt 3’	5’ gat cca ggc ctt ttg ttg aa 3’
Col 1a1	NM_007742	5' cat gtt cag ctt tgt gga cct 3'	5' gca gct gac ttc agg gat gt 3'
Col 1a2	NM_007743	5' gca ggt tca cct act ctg tcc t 3'	5' ctt gcc cca ttc att tgt ct 3'
Col 5a1	NM_015734	5' tga att caa gcg tgg gaa act 3'	5' ccg cag gaa ggt cat ttg tac 3'
Col 5a2	NM_007737	5' gaa agg ctg gtg atc aag gt 3'	5' ttt ctc ccc gag gtc cta at 3'
Col 5a3	NM_015719	5' ctt gcc gta cta gtg tc 3'	5' cct ctc ctc cct cgt ctt c 3'
MAPK1	NM_011949	5' aag aac tca ttt ttg aag aga ctg c 3'	5' ctc tga gcc ctt gtc ctg a 3'
Integrin B1	NM_010578	5' tgg caa caa tga agc tat cg 3'	5' atg tcg gga cca gta gga ca 3'
Fibronectin-1	NM_010233	5' cgg aga gag tgc ccc tac ta 3'	5' cga tat tgg tga atc gca ga 3'
MMP-2	NM_008610	5’gtg gga caa gaa cca gat cac 3’	5’ gcatcatccacggtttcag 3’
MMP-14	NM_008608	5' aac ttc gtg ttg cct gat ga 3'	5' ttt gtg ggt gac cct gac tt 3'
TGFB 1	NM_011577	5' tgg agc aac atg tgg aac tc 3'	5' cag cag ccg gtt acc aag 3'
TGFB2	NM_009367	5' tgg agt tca gac act caa cac a 3'	5' aag ctt cgg gat tta tgg tgt 3'
TGFB3	NM_009368	5' ccc tgg aca cca att act gc 3'	5' tca ata taa agg ggg cgt aca 3'
